# Supplementary material for: NPC1 enables cholesterol mobilization during long‐term potentiation that can be restored in Niemann–Pick disease type C by CYP46A1 activation
Source: EMBO Rep. 2019 Sep 18;20(11):e48143. doi: 10.15252/embr.201948143 (PMC6832102; doi:10.15252/embr.201948143)
Supplement: Supplementary file 1 — Appendix [file EMBR-20-e48143-s001.pdf]

APPENDIX

NPC1 enables cholesterol mobilization during Long-Term-Potentiation that can be restored in Niemann-Pick disease type C by CYP46A1 activation

Daniel N. Mitroi, Guadalupe Pereyra-Gómez, Beatriz Soto-Huelin, Fernando Senovilla, Toshihide Kobayashi, Jose A. Esteban and María Dolores Ledesma

TABLE OF CONTENTS **PAGE**

Appendix Figure S1. Immunoelectronmicroscopy against NPC1 2

Appendix Figure S2. Purity assessment of synaptosomal preparations 2

Appendix Figure S3. Biochemical analysis of the cLTP induction in synaptosomes 3

Appendix Figure S4. NPC1 levels in synaptosomes 4

Appendix Figure S5. Levels of synaptic proteins in *NPC1*^{nmf164} mice 4

Appendix Figure S6. Mobility and expression of GFPwtNPC1 and GFP D1005G NPC1 in hippocampal slices from wt mice using Sindbis virus 5

Appendix Figure S7. Cholesterol-specific staining of mCherry-D4 5

Appendix Figure S8. Paired pulse facilitation in NPC1^{nmf164} mice 6

Appendix Figure S9. Immunofluorescence against NPC1 in EFV treated neurons 6

Appendix Figure S10. EFV improves motor abilities and neuronal survival in NPC1^{nmf164} mice 7

Appendix Figure S11. EFV does not correct liver pathology in NPC1^{nmf164} mice 8

Appendix Figure S12. NPC2 levels in synapses 9

Appendix Fig S1

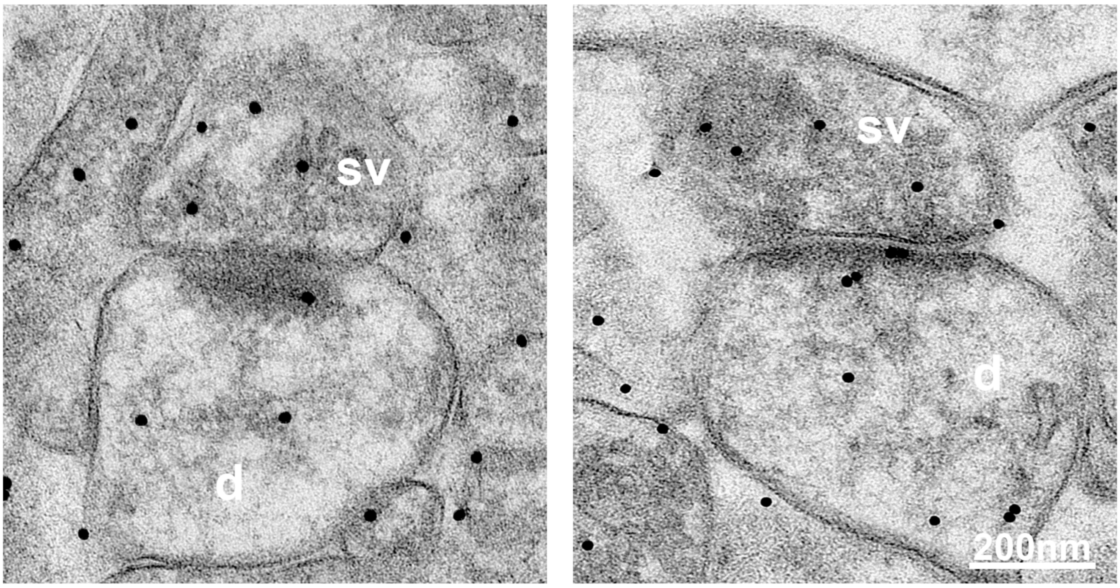

Appendix Figure S1. Immunoelectronmicroscopy against NPC1. Electron microscopy image of immunogold labelling against NPC1 in the CA1 hippocampal region of a wt mouse using the antibody rabbit monoclonal abcam #ab134113 (d-dendrite, sv-synaptic vesicles).

Appendix Fig S2

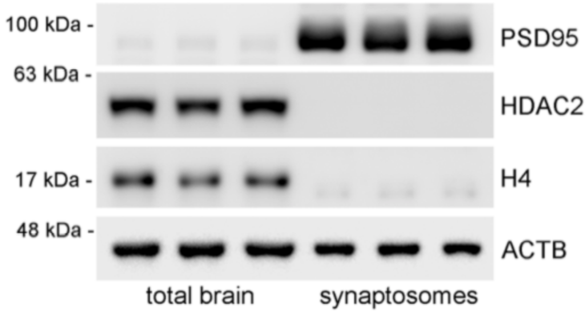

Appendix Figure S2. Purity assessment of synaptosomal preparations. Western blots against the postsynaptic marker PSD95, the nuclear markers histones HDAC2 and H4 and against actin-β (ACTB) in total brain and synaptosomal extracts from wt and *NPC1*^{nmf164} mice.

Appendix Fig S3

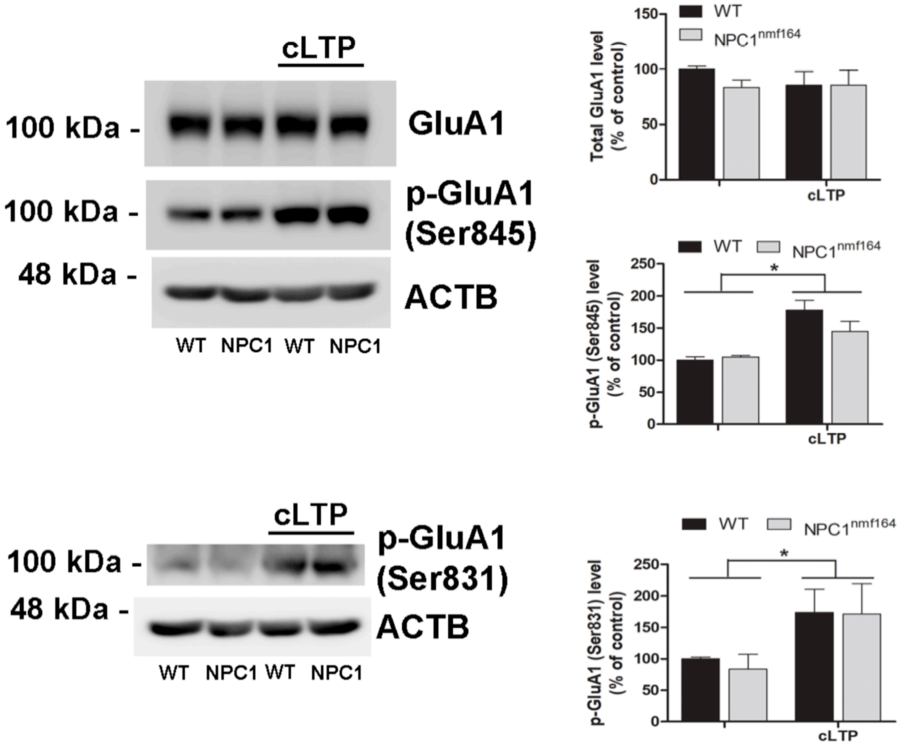

Appendix Figure S3. Biochemical analysis of the cLTP induction in synaptosomes. Western blots against GluA1 using antibodies insensitive to phosphorylation or detecting phosphorylation in Serines 845 and 831, which are indicative of GluA1 activation, in synaptosomes from wt and $NPC1^{nmf164}$ mice in which cLTP was induced or not. Graph shows the mean ± SEM of Ser845- (n=3 mice, 10 week-old, 2-way ANOVA, p = 0.0217) and Ser831-phosphorylated GluA1 (n=3 mice, 10 week-old, 2-way ANOVA, p = 0.0400) with respect to total non-phosphorylated GluA1.

Appendix Figure S4

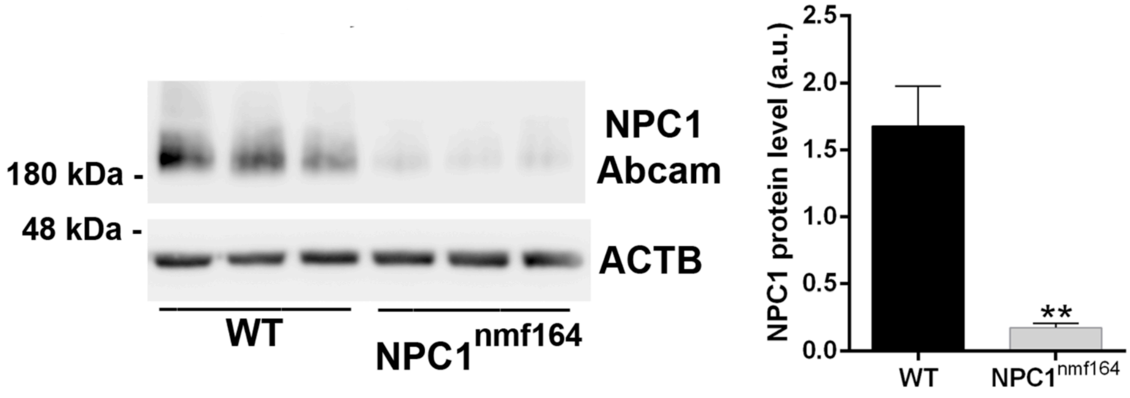

Appendix Figure S4. NPC1 levels in synaptosomes. Western blots against NPC1 using the abcam antibody #ab134113 and against actin-β (ACTB) in synaptosomes from wt and *NPC1^{nmf164}* mice. Graphs show mean ± SEM NPC1 level normalized to ACTB in arbitrary units (n=3 mice, 3 month-old, unpaired Student *t* test, p= 0.0075).

Appendix Figure S5

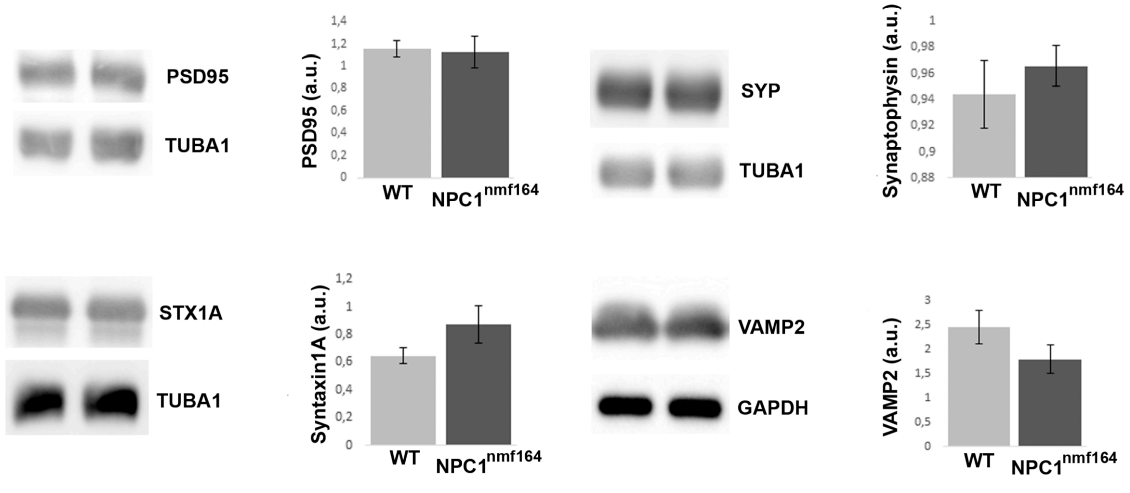

Appendix Figure S5. Levels of synaptic proteins in *NPC1^{nmf164}* mice. Western blots against the synaptic markers PSD95, Synaptophysin (SYP), Syntaxin 1 (STX1A) and VAMP2 and against the loading controls tubulin 1A (TUBA1) and GAPDH in synaptosomes from wt and *NPC1^{nmf164}* mice. Graphs show mean ± SEM level of the indicated synaptic proteins normalized to the corresponding loading controls in arbitrary units (n=3 mice, 3 month-old).

Appendix Figure S6.

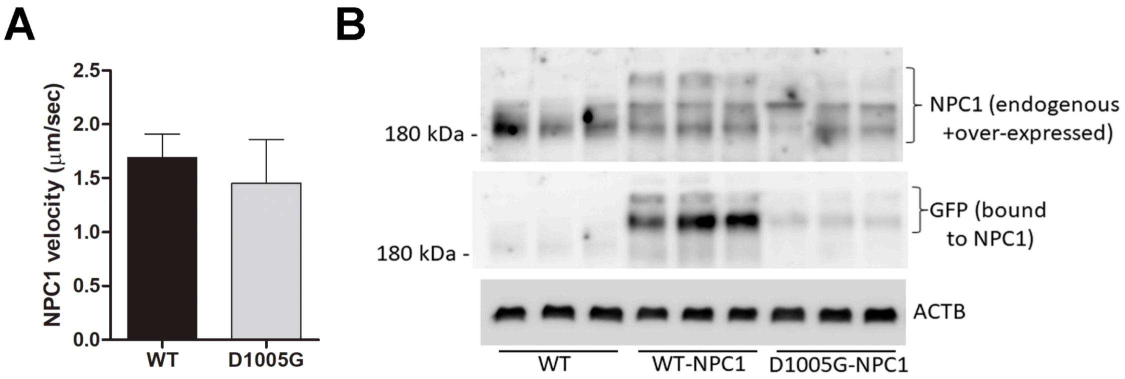

Appendix Figure S6. Mobility and expression of GFPwtNPC1 and GFP D1005G NPC1 in hippocampal slices from wt mice using Sindbis virus.

A. Graph shows the mean ± SEM (n ≥ 30 particles) velocity in µm/second of the GFPwtNPC1 or GFP D1005G NPC1 particles (see supplementary movies).

B. Western blots against NPC1, GFP or ACTB in hippocampal neuronal cultures from wt mice non infected or infected with Sindbis virus expressing GFPwtNPC1 or GFP D1005G NPC1.

Appendix Figure S7

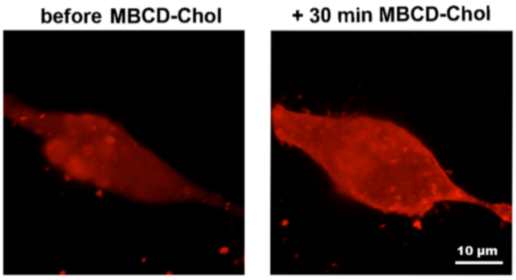

Appendix Figure S7. Cholesterol-specific staining of mCherry-D4. Representative fluorescence images of a cultured hippocampal neuron from wt mice transfected with mCherry-D4 before and after incubation with cholesterol complexed with methyl-β-cyclodextrin; Z-stacks are shown.

Appendix Figure S8

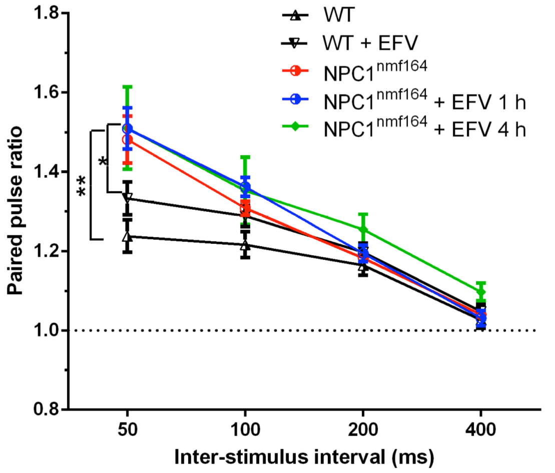

Appendix Figure S8. Paired pulse facilitation in NPC1^{nmf164} mice. Paired pulse facilitation in hippocampal slices from wt and NPC1^{nmf164} mice incubated or not with 20μM EFV at the indicated times (n=4 slices per condition from 2 wt and 2 NPC1^{nmf164} mice, 10 week-old, 2-way ANOVA, p = 0.0098)

Appendix Figure S9.

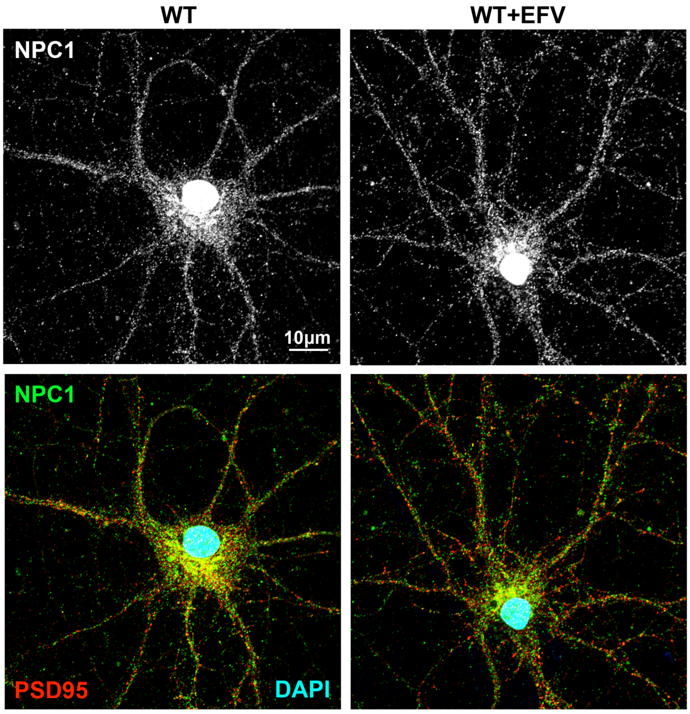

Appendix Figure S9. Immunofluorescence against NPC1 in EFV treated neurons. Single grey-scale and coloured merged images of immunofluorescence signals against NPC1 or against NPC1 and PSD-95, in a cultured hippocampal neuron from wt mice treated or not with 20μM EFV. DAPI stains cell nuclei.

Appendix Figure S10

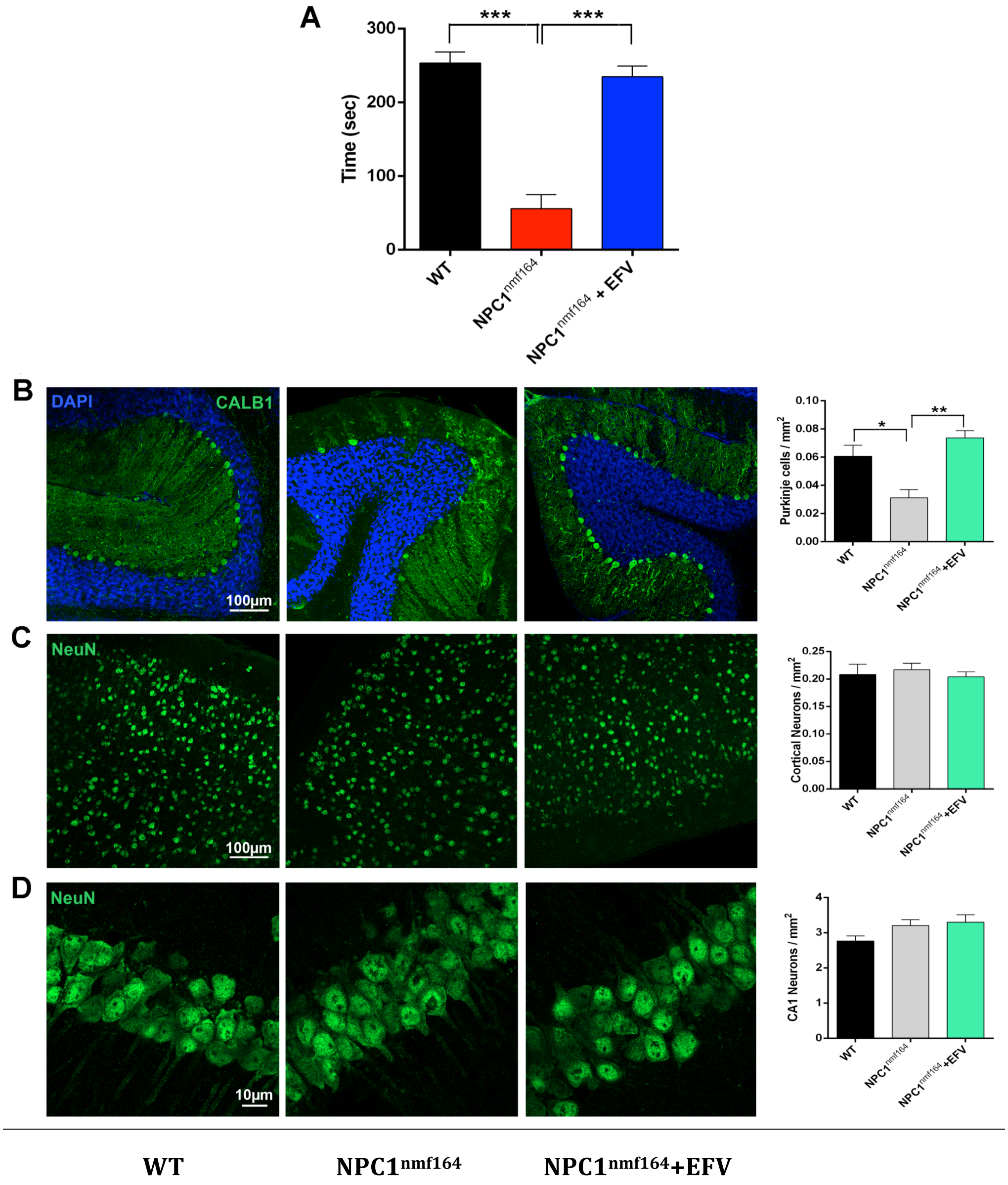

Appendix Figure S10. EFV improves motor abilities and neuronal survival in NPC1^{nmf164} mice. A. Mean ± SEM time spent on the rod in the Rotarod performance test in wt and NPC1^{nmf164} mice treated or not with 0.09mg/kg/day EFV (n=6 mice, 3-month old, one-way ANOVA, $p < 0.0001$). **B, C, D**. Neuronal death analysed by immunocytochemistry of calbindin in the Purkinje cells in the cerebellum (A) and of NeuN in cortical (C) and hippocampal (D) neurons. Graphs show mean ± SEM number of neurons per area (n= 3 mice, 14-week old, one-way ANOVA, $p_{\text{Purkinje cells}} = 0.0049$)

Appendix Figure S11

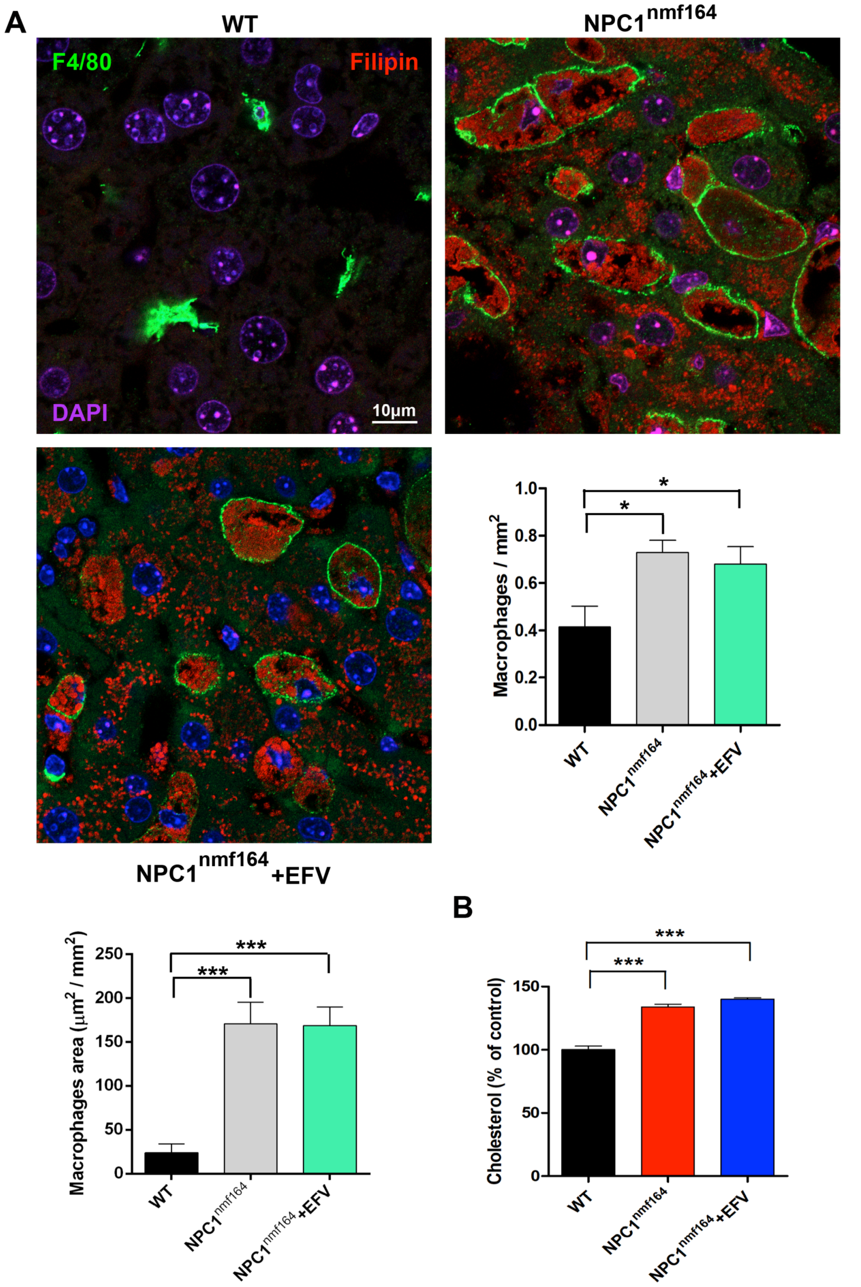

Appendix Figure S11. EFV does not correct liver pathology in NPC1^{nmf164} mice. A. Liver from wt and NPC1^{nmf164} mice treated or not with 0.09 mg/kg/day EFV stained for the specific macrophage marker F4/80 and for filipin. Graphs show mean ± SEM number and area of macrophages (n= 7 confocal images, one-way ANOVA, p_{number} = 0.0139, p_{area} < 0.0001). **B**. Mean ± SEM cholesterol levels in liver extracts from wt and *NPC1^{nmf164}* mice measured by enzymatic assays and expressed as percentage of wt mice (n= 3 technical replicates, one-way ANOVA, p < 0.0001).

Appendix Figure S12

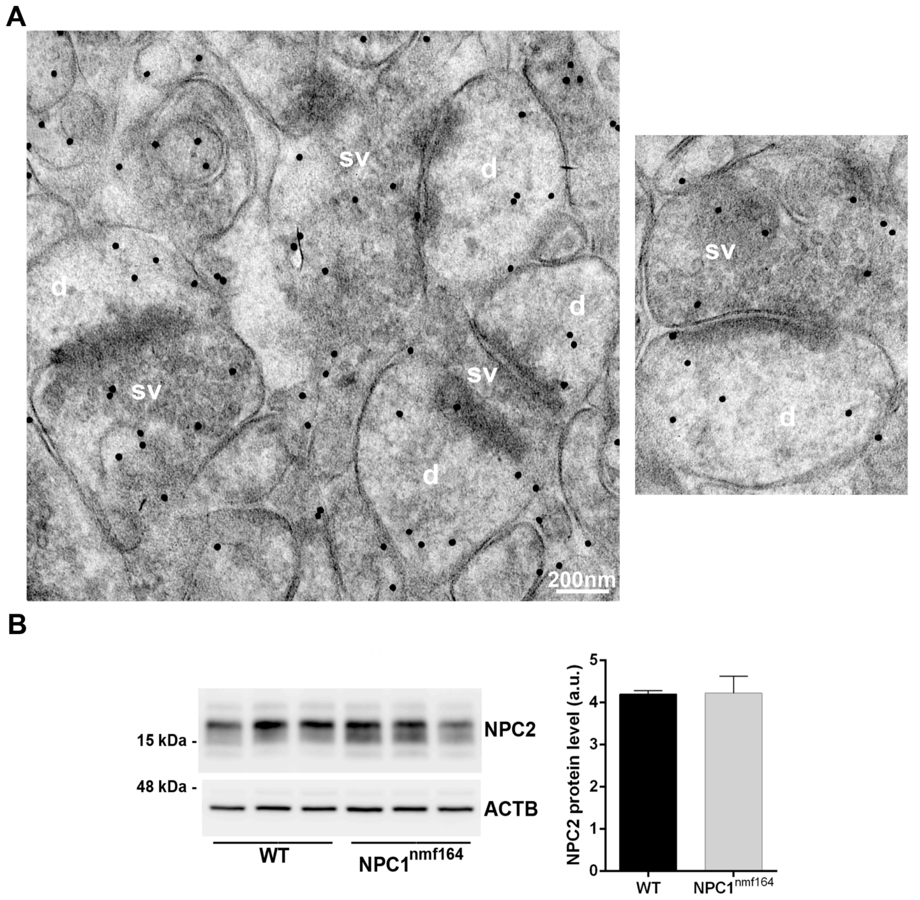

Appendix Figure S12. NPC2 levels in synapses. A. Electron microscopy image of immunogold labelling against NPC2 in the CA1 hippocampal region of a wt mouse (d-dendrite, sv-synaptic vesicles). **B.** Western blots against NPC2 and actin-β (ACTB) in synaptosomes from wt and *NPC1^{nmf164}* mice. Graphs show mean ± SEM NPC2 level normalized to ACTB in arbitrary units (n=3 mice, 3-month old).
