# Supplementary material for: NPC1 enables cholesterol mobilization during long‐term potentiation that can be restored in Niemann–Pick disease type C by CYP46A1 activation
Source: EMBO Rep. 2019 Sep 18;20(11):e48143. doi: 10.15252/embr.201948143 (PMC6832102; doi:10.15252/embr.201948143)
Supplement: Supplementary file 2 — Movie EV1 [file EMBR-20-e48143-s002.zip › Movie_EV1/Movie_EV1_Legend.docx]

**Movies EV1 (annex to Appendix Figure S3).** Representative videos of the mobility of GFPwtNPC1 in neuronal processes of hippocampal slices from wt mice infected with Sindbis virus expressing GFPwtNPC1.
