# Supplementary material for: NPC1 enables cholesterol mobilization during long‐term potentiation that can be restored in Niemann–Pick disease type C by CYP46A1 activation
Source: EMBO Rep. 2019 Sep 18;20(11):e48143. doi: 10.15252/embr.201948143 (PMC6832102; doi:10.15252/embr.201948143)
Supplement: Supplementary file 3 — Movie EV2 [file EMBR-20-e48143-s003.zip › Movie_EV2/Movie_EV2_Legend.docx]

**Movie EV2 (annex to Appendix Figure S3).** Representative video of the mobility of GFP D1005G NPC1 in neuronal processes of hippocampal slices from wt mice infected with Sindbis virus expressing GFP D1005G NPC1.
