# Supplementary figures and images for: NPC1 enables cholesterol mobilization during long‐term potentiation that can be restored in Niemann–Pick disease type C by CYP46A1 activation
Source: EMBO Rep. 2019 Sep 18;20(11):e48143. doi: 10.15252/embr.201948143 (PMC6832102; doi:10.15252/embr.201948143)

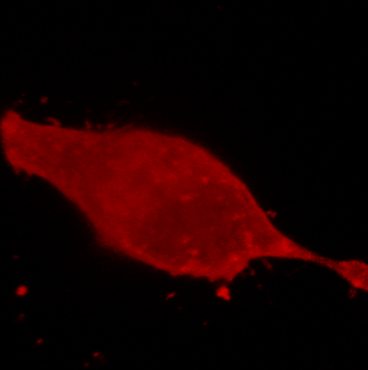

Supplement: Supplementary file 4 — Source Data for Appendix [file EMBR-20-e48143-s007.zip › Source_data_Figure_S7/S7,_+_30_min_MBCD-Chol_Volume_Snapshot.tif]

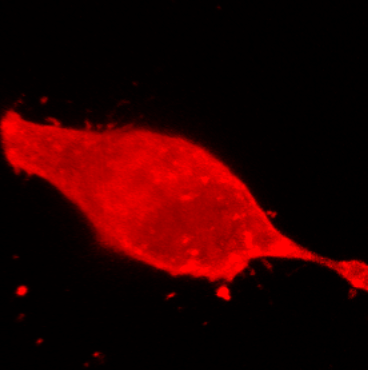

Supplement: Supplementary file 4 — Source Data for Appendix [file EMBR-20-e48143-s007.zip › Source_data_Figure_S7/S7,_+_30_min_MBCD-Chol_Volume_Snapshot_increased_intensity.tif]

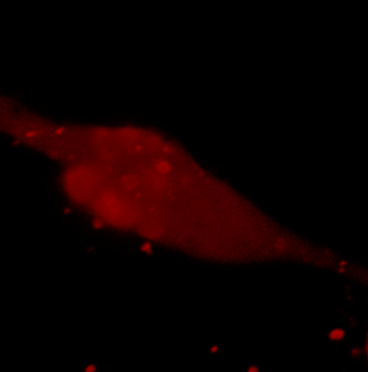

Supplement: Supplementary file 4 — Source Data for Appendix [file EMBR-20-e48143-s007.zip › Source_data_Figure_S7/S7,_before_MBCD-Chol_Volume_Snapshot.tif]

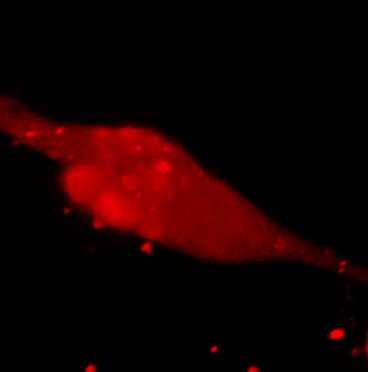

Supplement: Supplementary file 4 — Source Data for Appendix [file EMBR-20-e48143-s007.zip › Source_data_Figure_S7/S7,_before_MBCD-Chol_Volume_Snapshot_increased_intensity.tif]

\* = lanes showed in image 4 B

Full unedited gel for Figure 4 B

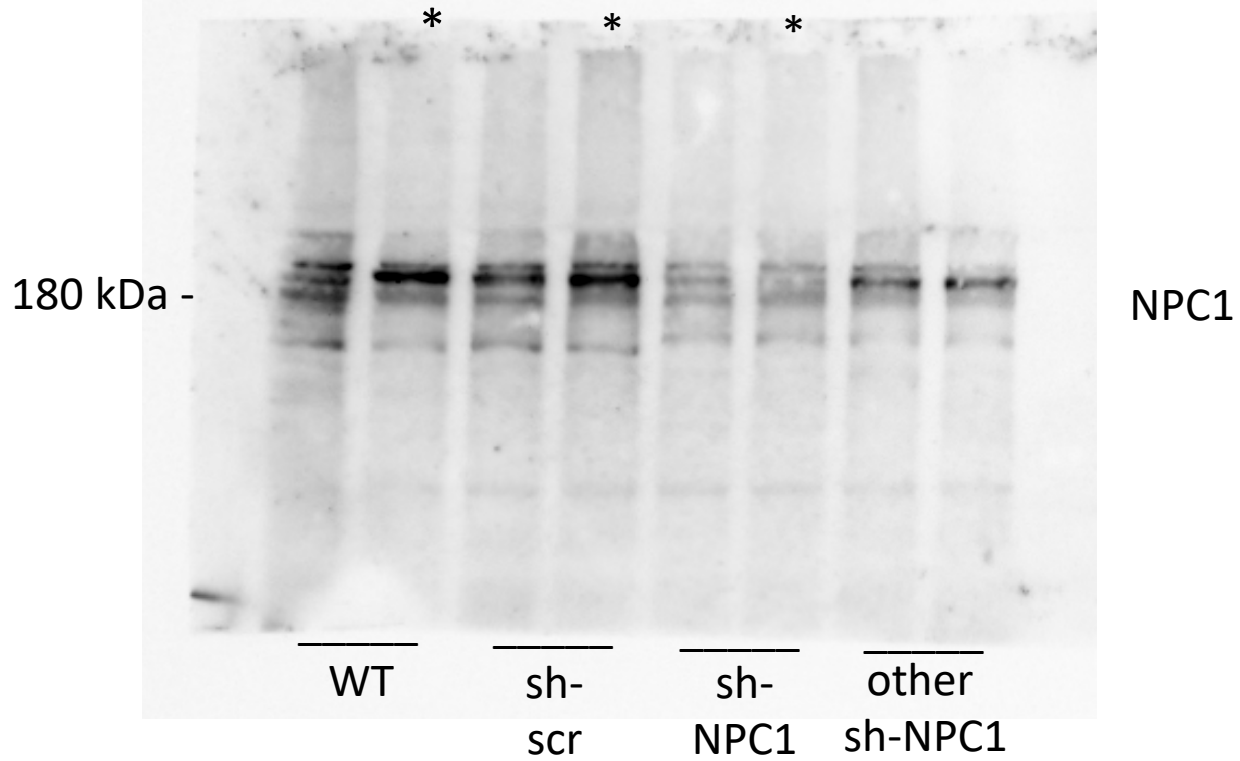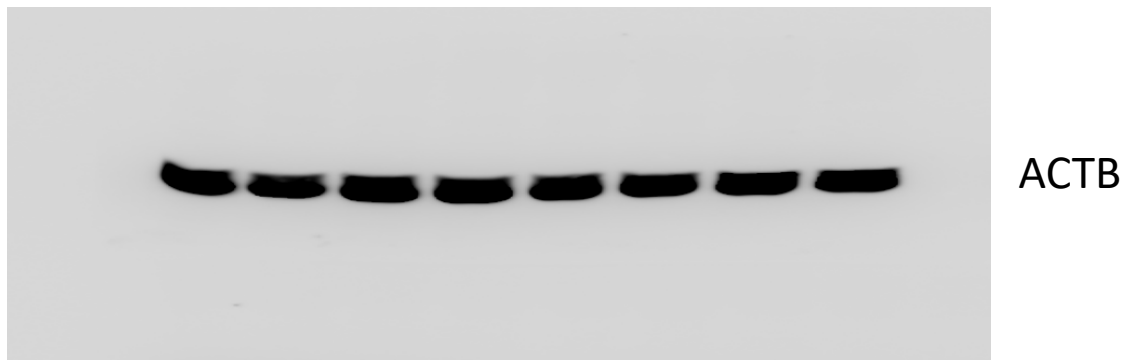

Full unedited gel for Figure 4 D

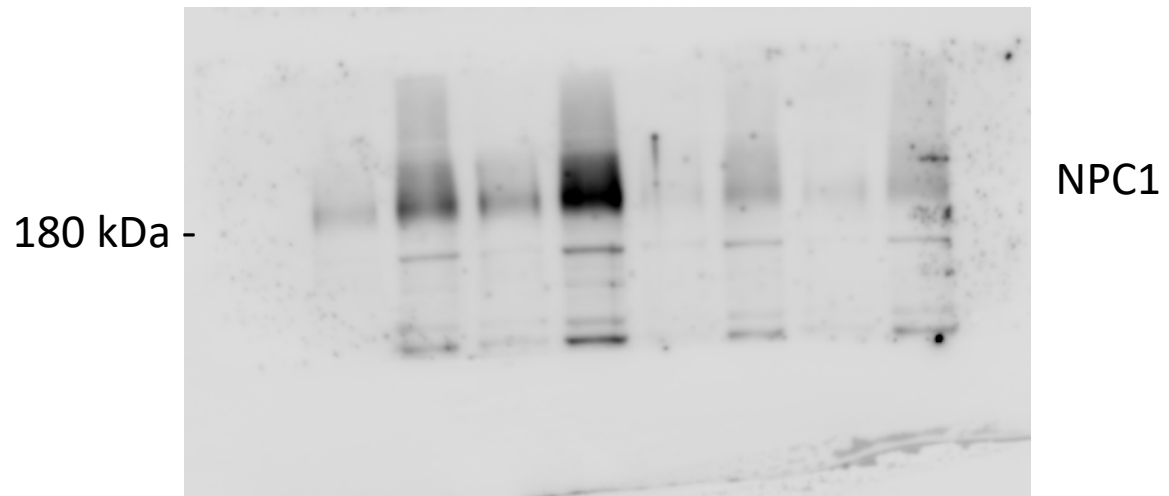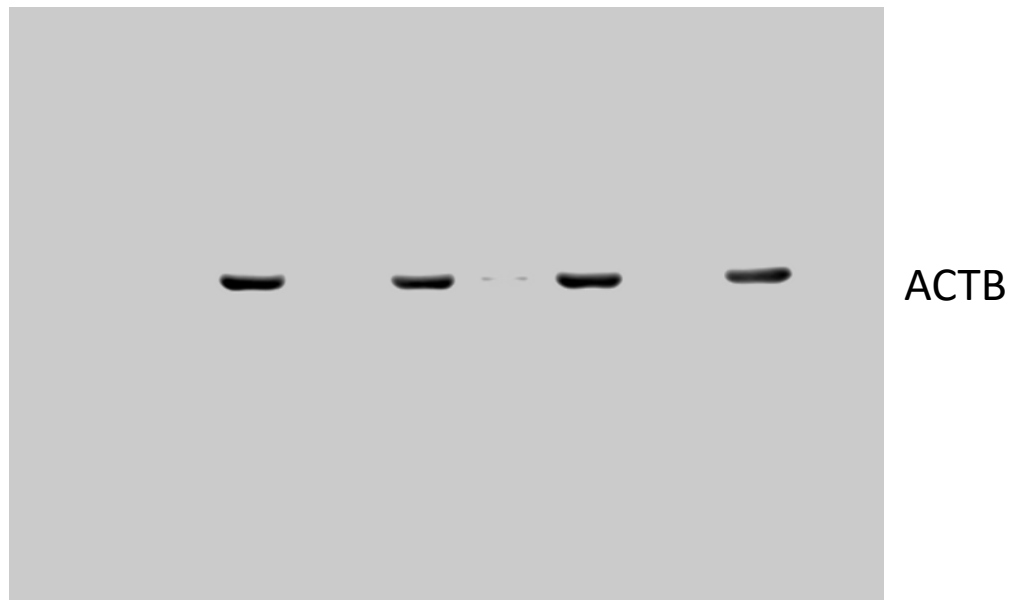

Supplement: Supplementary file 7 — Source Data for Figure 4 [file EMBR-20-e48143-s005.pdf]
